# Supplementary figures and images for: Activation and inhibition of the C-terminal kinase domain of p90 ribosomal S6 kinases
Source: Life Sci Alliance. 2023 Feb 20;6(5):e202201425. doi: 10.26508/lsa.202201425 (PMC9941302; doi:10.26508/lsa.202201425)

Fruergaard et al. Source data related to Figure 3

Figure 3A

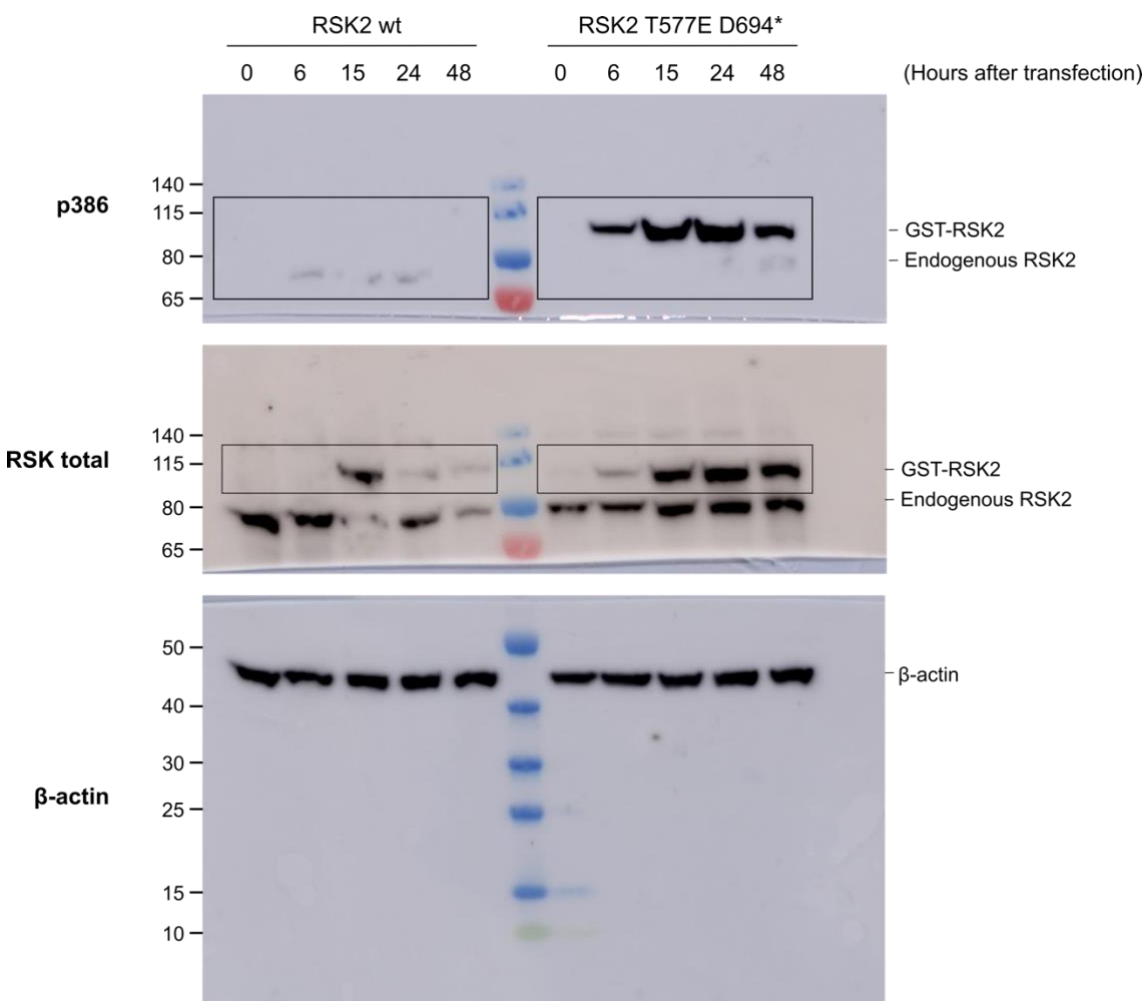

**Figure 3B**

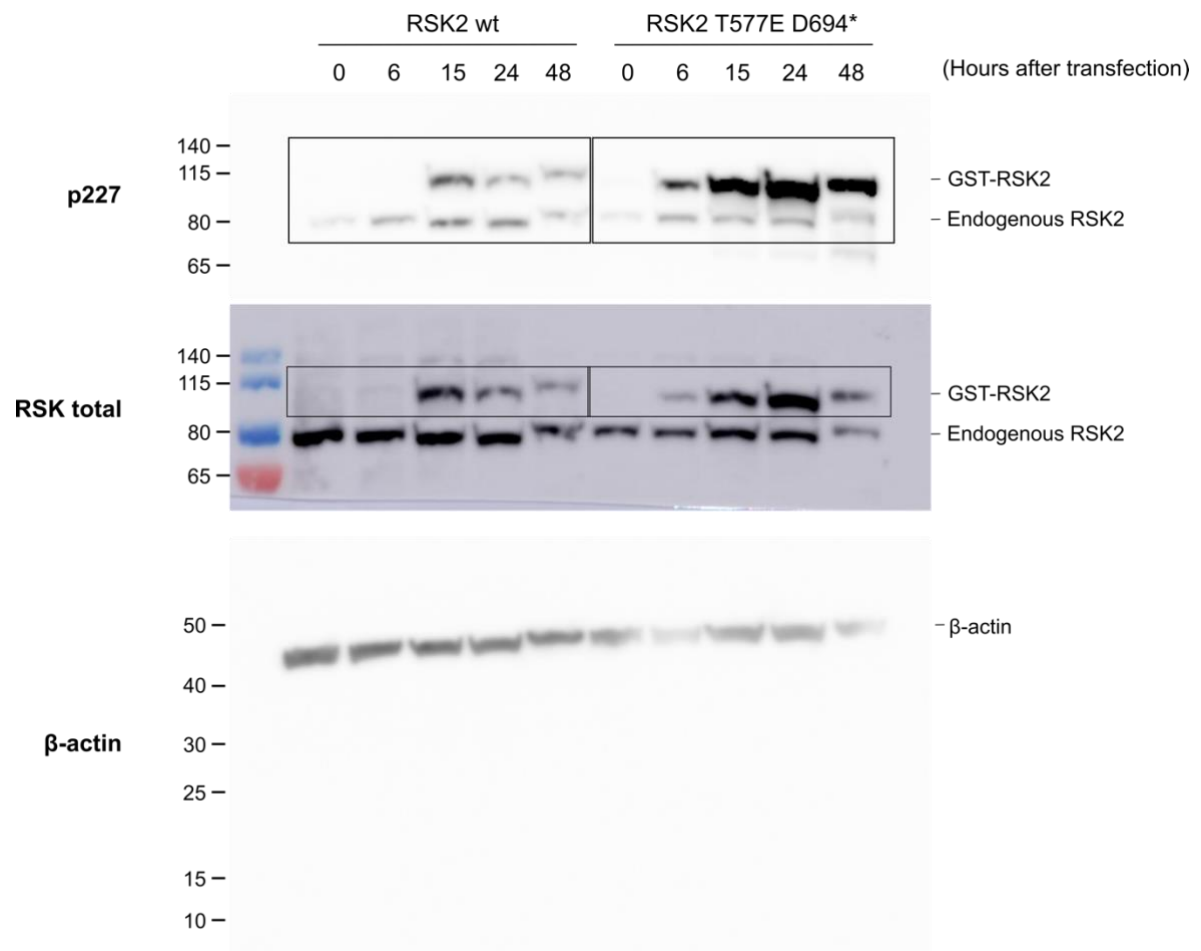

Supplement: Supplementary file 1 [file LSA-2022-01425_SdataF3.1.pdf]
